# Exploring the Learning Process for Neonatology Fellows of Giving Serious News on a Virtual Platform

**DOI:** 10.7759/cureus.76087

**Published:** 2024-12-20

**Authors:** Sandra Aziz, Samantha Hurst, Krishelle L Marc-Aurele

**Affiliations:** 1 Division of Quality of Life and Pediatric Palliative Care, Department of Pediatrics, Stanford University, Palo Alto, USA; 2 Medical Anthropology, Herbert Wertheim School of Public Health and Human Longevity, University of California San Diego, La Jolla, USA; 3 Pediatrics and Neonatology, University of California San Diego, La Jolla, USA

**Keywords:** communication, giving serious news, interviews, learning, neonatology, palliative care, simulation, virtual platform

## Abstract

Background

Since 1998, the Accreditation Council for Graduate Medical Education has highlighted the importance of teaching palliative care skills, yet neonatology fellows sometimes receive varied and inadequate training on these topics. As this study was conducted during the COVID-19 pandemic when our medical education curriculum was still mostly remote, our aim was to explore how neonatology fellows learned to give serious news with the use of a virtual platform.

Objectives

The aim of this study was to explore, through qualitative interviews after virtual simulations, the individual learning experiences of giving serious news to a standardized patient (SP).

Methods

Between January and December 2022, neonatology fellows participated in quarterly two-hour virtual sessions with an SP. At each session, two fellows practiced giving serious news with an SP, and after the session, both fellows were interviewed about their experiences. Analysis of interviews was conducted with a team-based approach using qualitative data analysis software.

Results

The following four major themes were identified: types of training, role-playing, virtual platform, and lessons learned. Simulation training was found to be beneficial in the learning and practicing of giving serious news. The virtual environment was viewed as a safe learning environment. Approximately half of the participants stated that the virtual learning environment may be more comfortable compared with in-person simulations.

Conclusion

Online simulation sessions can serve as a safe, valuable, and comfortable platform to teach and practice skills for giving serious news.

## Introduction

Simulation-based medical education has been utilized to train learners at all levels to disclose serious news [1-6], and neonatology fellows sometimes receive variable and incomplete training in communication and palliative care skills [7-8]. According to a 2021 study determining whether palliative care education is effective in 24 different U.S. neonatology programs, most of the responding fellows (80%) reported being uncomfortable or only somewhat comfortable with essential areas of palliative care [9]. We thus created a simulation curriculum utilizing a standardized patient (SP) with whom neonatology fellows could practice giving serious news. Because our curriculum was mostly still remote due to the COVID-19 pandemic, we designed the simulation session to be virtual. The aim of this study was to investigate the learning process for neonatology fellows of giving serious news on a virtual platform.

This article was previously presented as a meeting abstract at the 3rd Annual Neonatal-Perinatal Research Conference, Alamo, Texas, October 2022.

## Materials and methods

Qualitative interviews

Following the simulation sessions, nine fellows who gave serious news to the SP were asked to participate in a one-on-one qualitative interview comprising 10 questions (Table 1). Using the Zoom Platform, a total of nine interviews were completed between January and December 2022. Interview guides were designed in a semi-structured, open-ended format to explore each participant’s individual learning process. The majority of the interviews were completed in approximately 20-30 minutes. All interviews were digitally recorded and transcribed using Otter.ai™ 2023. To ensure accuracy, each transcript was proofread against the audio file prior to initiating coding and content analysis.

**Table 1:** Post-session semi-structured open-ended interview guide

Interview Questions
The following questions will be asked to the fellow playing the provider following the virtual simulation session. They are focused on addressing how the virtual session impacts simulation learning and to explore how this particular simulation session helps the participating fellow learn how to give serious news.
How have you been trained to give serious news in the past? Can you give me any meaningful examples?
What did you feel about delivering serious news in today’s session?
How did the Zoom online platform in today’s session influence your individual learning process?
How helpful was reviewing the instruction video prior to giving serious news in this simulation session?
If you were unable to review the instruction video, what other materials would be helpful in preparation for learning this material?
If you were an observer at a previous session, how did that experience facilitate your delivery of serious news in today’s session?
What is one important lesson you learned in today’s session that will help you deliver serious news in the future?
What did you think about the debrief session? Please give me some examples.
What would make this session more helpful for you, specifically, to learn how to give serious news?
How do you believe this simulation session would be different if it were done in person?

Qualitative analysis

A conventional qualitative content analysis approach was employed with attention to the aims of the study and direct interview questions. Preliminary cycles of open and focused coding were conducted with a qualitative expert (S.H.) and primary investigator (S.A.). From this review, a content codebook was created by S.A. to identify contextualized segments of data that corresponded to targeted questions. After the final coding schema was determined, all interview transcripts were uploaded to Quirkos (Quirkos Cloud Ltd, Edinburgh, UK), a qualitative analysis program to facilitate the arrangement of coded excerpts into essential concepts and thematic results.

Because the number of interviews in this study was low and the analysis was qualitative, specific numbers and percentages were not employed to describe the data. A majority is meant to represent more than 50% but fewer than 75%, and, similarly, a minority represents less than 50% but more than 25%.

Simulation session

Nine neonatal-perinatal fellows in all years of training were recruited. During two-hour long sessions, two fellows were identified to give serious news to a trained SP. Prior to each session, the participating fellows were each sent a scenario (Table 2), a recorded didactics lecture covering basic concepts of giving serious news, and a skills checklist. In one scenario, the SP is pregnant at 21 0/7 weeks’ gestation, and preterm delivery at this gestational age portends the expected death of the infant. In the other scenario, the SP is pregnant at 23 0/7 weeks’ gestation. The slightly older gestational age was chosen to provide additional complexity to the scenario. Infants born at this gestational age can survive with a high risk of neurodevelopmental impairment. Each scenario was approximately 30 minutes. Fellows were given the opportunity to stop the interaction and ask for assistance at any time. Following the interaction, all attendees, along with the SP, participated in a debrief session for another 30 minutes. All actions were repeated for the second scenario, resulting in a two-hour-long simulation.

**Table 2:** Simulation scenario In one scenario, the SP is pregnant at 21 0/7 weeks’ gestation, and preterm delivery at this gestational age portends expected death of the infant. In the other scenario, the SP is pregnant at 23 0/7 weeks’ gestation to provide additional complexity. Infants born at this gestational age can survive with high risk of neurodevelopmental impairment. MFM, maternal-fetal medicine

Instructions to the Neonatology Fellow
We ask that you treat the scenario as real and immerse yourself in the role. You are in a hospital setting and can call upon resources you would normally have.
Your patient is a 33-year-old G1P0 female at 21 0/7 (OR alternatively 23 0/7) weeks’ gestation by IVF dating who was sent from the clinic for suspected preterm labor. The patient’s partner has tested positive for COVID-19 and she is currently pending results (no symptoms). Therefore, she will need to communicate with medical providers via video communication.
Vital signs
Temperature: 37.2°C
Pulse: 106 beats per minute
Respiration: 18 breaths per minute
Blood pressure: 120/62
Estimated fetal weight: 250 grams (or alternatively 500 grams)
You are to tell her the difficult news.
Fellow: You are asked to talk with the patient because her contractions are becoming more frequent along with progressive cervical dilation. Delivery appears imminent. You are asked by your MFM colleagues to consult because it is not clear what her goals of care are for this baby. As you know, survival for this baby is 0% (OR alternatively 56%) based on the last six years of data. If the baby does survive, there is a high risk of neurodevelopmental impairment.
You have 15 minutes to perform these tasks. Once you have told her the tough news, you will excuse yourself when appropriate (facilitator will update you).
Tips for session: Use a wired headset to minimize audio issues
If you are the “physician” in the role-playing scenario: Look at camera when talking to the patient
If you are not the “physician” in the role-playing scenario:
Update your Zoom “name” to be your study number
Hide video during scenario
Select “Hide Non-Video participants” by clicking on three dots at right-hand corner of the non-video participant
Take notes on what is going well during scenario
Restart video for discussion part of scenario so facilitator can see you

This single-center pilot study protocol #210130XX, “Pilot Study: Evaluating the Feasibility of Role-Playing for Fellows to Practice Skills Giving News Using a Virtual Platform,” was reviewed by the University of California San Diego (UCSD) Human Research Protections Program and certified as exempt from IRB review [KM1] under 45 CFR 46.104(d) category 2 on February 8, 2021.

## Results

Four overlapping themes with corresponding sub-themes emerged from the analysis and are identified as follows (Table 3).

**Table 3:** Representative quotes for each of the four overlapping themes with corresponding sub-themes

Themes: Subthemes	Quotes
Types of training: prior to study and training value	“I had the opportunity to get some sort of foundation in how to communicate with families and to deliver serious or difficult news at various stages of my training.” (second-year fellow)
	“Some attendings were really good at connecting with the families and getting on their level and being empathetic when they were giving bad news. Some attendings in residency maybe didn't show enough empathy given the level of the situation. So I was able to kind of pick and choose things that I liked that I could carry forward in how I present bad news.”(second-year fellow)
Role-playing: provider role and observer role	“I think it's really hard to separate your feelings from the scenario because it's not about you…it's really hard to be that uncomfortable…to acknowledge that we can't fix everything…And I think it's hard to be with somebody in that much pain, and let them have that space to be in pain…you have to make sure your feelings are not overwhelming their feelings.” (second-year fellow)
	“It’s one of those things that you remember that it’s five minutes out of your day or 30 minutes out of your day, but it is a lifetime for the family.” (first-year fellow)
	“I think that being like a fly on the wall is always a helpful learning experience because you’re able to experience the whole scenario without the anxiety, and stressor of being directly in it.” (first-year fellow)
Virtual platform: advantages, disadvantages, and comparison to in-person	“It was beneficial because it allows you to bring more people together and get more perspectives from everybody. I think that it's a reasonable thing that happens nowadays…we have to do these things on telephones sometimes. I think that it adequately reflected the kind of situation and still definitely able to get involved in this scenario and feel the emotion.” (first-year fellow)
	“I think if it was done in person, I think it would definitely be a little bit more realistic…I do feel like it would be a little bit more awkward, though, because everyone else would be watching. I actually feel like I might not even perform as well or do my best job.” (third-year fellow)
	“You could almost say that Zoom was almost a little protective, too.” (first-year fellow)
Lessons learned: simulation session and debrief session	“I'm actually really happy that this is the first time I encountered that conversation, as opposed to in real life…I learned to allow for time for emotion and that giving enough time for people to process is really important for them to actually hear and feel and understand.” (first-year fellow)
	“I think it’s helpful to practice with a standardized patient because you can get feedback from them. Whereas you can’t get feedback from a parent.” (second-year fellow)

Types of training: prior to study and training value

A majority of the fellows recall observing other providers having serious conversations during their medical school and/or residency training. Approximately half of the participants received formal training in giving serious news during medical school, and even fewer during residency. One-third of the participants had prior experience from a palliative care rotation. All participants observed a prenatal consultation during orientation and attended palliative care lectures as part of their fellowship program orientation.

A majority of the participants believed that their former training, specifically observing others, was useful to them since they were able to identify, adopt, and adapt those skills to their own practice.

Role-playing: provider role and observer role

All participants expressed that having to give serious news to a patient/family was nerve-wracking and anxiety-producing. Around half of the participants expressed that comforting grieving families was challenging and felt that it was difficult to hold space for and bear witness to a parent's pain and sadness. One-third of the participants mentioned that they felt uncomfortable being unable to offer a patient or family more in a situation where an infant is not likely to survive, and one fellow mentioned that it was “hard not to feel like I failed them in some way too.”

Participants stated that being an observer was beneficial since they were able to pick up useful communication skills without additional performance anxiety. Approximately half of the participants mentioned that they learned best by being a “fly on the wall,” and a minority of the fellows added that repeated exposure to these conversations helped “cement” skills and techniques. Furthermore, observing a more senior-level fellow allowed them the opportunity to appreciate advancing conversation skills.

Virtual platform: advantages, disadvantages, and comparison to in-person sessions

A majority of the participating fellows admitted that the virtual platform made it often difficult to connect with the SP and did not provide a realistic representation of in-person encounters. One-third of the fellows described difficulty interpreting body language and energy. A minority of the participants also mentioned the risk of experiencing technical complications.

However, all of the participants thought that utilizing the virtual platform was useful. They felt that the simulation reflected the increased use of telemedicine due to the COVID-19 pandemic. Approximately half of the participants mentioned that the virtual platform allowed for multiple people to share their perspectives and provide immediate feedback in verbal and written format. Approximately half of the participants shared that they found the virtual platform to be more comfortable and safe than in-person simulations. For the fellow giving serious news to the SP, the virtual simulation sessions gave the illusion of being alone with the SP because of the option to hide non-video participants. A third of the participants said that in-person simulations can be “awkward” or “intimidating” because of increased awareness of being watched directly by multiple observers in the same room. The majority of the fellows believed that the virtual platform was an effective way to learn how to give serious news.

Lessons learned: simulation session and debrief session

The majority of the fellows were able to recall the communication skills they learned. They shared that they learned the importance of speaking in a clear way and providing information in small pieces. Furthermore, around half of the participants emphasized learning the importance of supportive silence. A minority of the fellows added that they were becoming more comfortable with giving serious news and appreciated having the chance to practice skills and phrasing in a safe, simulated environment.

The majority of the fellows appreciated receiving feedback during the debrief sessions. Feedback that fellows received from the debrief sessions included that the SP often wanted more time and space to process the serious news, that supportive silence was not uncomfortable for the SP, and that the SP felt comforted when fellows validated the SP’s emotions. Fellows expressed that receiving positive feedback served as excellent reinforcement of newly acquired skills. A few fellows mentioned that receiving feedback directly from the SP was vital.

## Discussion

This study explored how neonatology fellows learn to give serious news in a virtual simulation environment. The following four major themes emerged: types of training, role-playing, virtual platforms, and lessons learned. Our findings highlight the potential of virtual simulations to provide a safe, comfortable, and effective learning environment for practicing challenging communication skills.

One significant observation was the role of the virtual platform in fostering psychological safety. Participants frequently noted that the virtual environment reduced performance anxiety compared to in-person simulations. The option to hide non-video participants, combined with the comfort of engaging from home, creates a space where learners feel less self-conscious about being observed [10-11]. This aligns with prior studies demonstrating that psychological safety encourages learners to ask questions, admit mistakes, and focus on skill development without fear of judgment [12-14].

Participants also emphasized the value of clear communication strategies such as delivering information in manageable segments and employing supportive silence. The virtual format, while less realistic than in-person interactions, allowed fellows to rehearse these skills in a controlled, low-pressure environment. This aligns with findings in other studies on telemedicine and virtual education, where such platforms have been shown to effectively bridge gaps in experiential learning [15-17].

Importantly, the virtual platform reflects the growing integration of telemedicine in clinical practice [18-19]. The flexibility and accessibility of virtual simulations not only make them a practical alternative during pandemics or other disruptions but also position them as a complementary tool alongside in-person training [20-22]. Future curricula could benefit from a hybrid approach, where virtual simulations build foundational skills before transitioning to higher-stakes in-person scenarios.

Despite these strengths, this study has limitations. The virtual simulations were not analyzed to objectively document learned skills. The small, single-center sample and focus on neonatology fellows limit the generalizability of findings. Additionally, participants’ prior exposure to palliative care training may have positively influenced their experiences, underscoring the need for broader studies across diverse medical specialties.

## Conclusions

Virtual simulations offer a promising avenue for teaching communication skills, particularly in giving serious news. By creating a psychologically safe and comfortable environment, these platforms enable trainees to develop and refine essential skills. Future studies should explore the integration of virtual and in-person simulation curricula to enhance learning outcomes across medical education.
